# The effect of finasteride on the prostate gland in men with elevated serum prostate-specific antigen level

**DOI:** 10.1038/sj.bjc.6690670

**Published:** 1999-09

**Authors:** C A Coltman

**Affiliations:** Southwest Oncology Group, 14980 Omicron Drive, San Antonio, TX, 78245-3217, USA


*British Journal of Cancer* (1999) **81**(1), 184–186
© 1999 Cancer Research Campaign
Article no. bjoc.1999.0670

Letters to the Editor

### The effect of finasteride on the prostate gland in men with elevated serum prostate-specific antigen level

### Sir

We read with interest the report of Cote and colleagues (Cote et al, 1998) regarding the series of 52 men at risk of prostate cancer who received finasteride, or were observed and then biopsied after a 1-year period. The population of patients were at high risk for prostate cancer, despite an initial negative biopsy, by virtue of a mean baseline prostate-specific antigen (PSA) of 9.18 and 10.30 ng ml$^{-1}$ respectively, and 8/27 and 5/25 with prostatic intraepithelial neoplasia (PIN) on initial biopsy. Their results indicated no significant change in proliferation cell nuclear antigen (PCNA) index but a prostate cancer detection rate of 30% compared to 4% in patients treated with finasteride or controls respectively. For this reason, the authors conclude that, 'This study raises serious questions about the probable efficacy of finasteride in preventing prostate cancer'.

The authors' concern is of importance as the National Cancer Institute is currently sponsoring the Prostate Cancer Prevention Trial (PCPT) which is assessing the ability of finasteride to prevent prostate cancer in a population of 18 882 men who are without disease by virtue of a PSA $\leq$ 3.0 ng ml$^{-1}$ and a normal digital rectal examination. The rationale for the potential efficacy of finasteride in the prevention of prostate cancer has been described previously in detail (Gormley et al, 1995; Thompson et al, 1997). Although the findings of Cote are of interest, serious concerns regarding the study and its results should be understood.

While in the design of the PCPT the statistical assumptions called for a 90% power to detect a 25% reduction in incidence of prostate cancer, assuming a placebo-positive biopsy rate of 6% and a two-sided alpha of 0.05, it is unclear what study assumptions of the investigators led to a sample size of 52. It is certain, however, that small sample size studies can often lead to spurious results. For example, in the Cote study, if rather than 8/27 and 1/25 patients with positive biopsies, the rates were 7/27 and 1/25, the *P*-value would change from 0.025 to 0.051. This is even more dramatic when the patients with PIN are removed from the sample – the resulting patients had rates of 2/19 and 1/20 – a meaningless difference in prostate cancer rates.

Perhaps most of concern is the prostate cancer diagnosis rate among controls in Cote's study. In this group of patients with a mean PSA of 10.30 ng ml$^{-1}$, of whom five had PIN on pre-study biopsy, only one (4%) had a positive 1-year biopsy. This is most interesting as many authors in much larger series of patients have reported that rates of positive second sextant biopsies of patients with persistently elevated PSA have ranged from 13% to 26% (Keetch et al, 1994; Eskey et al, 1997; Niesel et al, 1997). If one looks for patients more akin to those of Cote, one group of men with PSAs > 10 had a 33% rate of positive-biopsy and another group of 48 men with high-grade PIN had a 47.9% rate of positive re-biopsy (Keetch et al, 1994; Raviv et al, 1996). The sum of these data suggest that the 30% prostate cancer detection rate in the very high-risk group of patients who were randomized to receive finasteride was to be expected. What is difficult to explain is the extraordinarily low rate of prostate cancer in the controls. One must assume that an unknown source of bias was operational as the most likely explanation or that this finding represents the play of chance attributable to the small study size.

Apropos to this, the recent publication of 4-year follow-up data of 3020 men randomized to placebo or finasteride found equivalent rates of cancer detection in both study arms (McConnel et al, 1998).

Evidence continues to develop that finasteride may have a preventive role in patients who are at risk of prostate cancer (Tsukmoyo et al, 1998). The results of the Prostate Cancer Prevention Trial will have enough power to address this extremely important issue.

*CA Coltman*
*Southwest Oncology Group,*
*14980 Omicron Drive, San Antonio,*
*TX 78245-3217, USA*

### REFERENCES

Cote RJ, Skinner EC, Salem CE, Mertes SJ, Stanczk FZ, Henderson BE, Pike MC and Ross RK (1998) The effect of finasteride on the prostate gland in men with elevated serum prostate-specific antigen levels. *Br J Cancer* **78**: 413–418

Eskey LA, Bare RL and McCullough DL (1997) Systematic 5-regional prostate biopsy is superior to sextant method for diagnosing carcinoma of the prostate. *J Urol* **157**: 199–203

Gormley GJ, Brawley O and Thompson I (1995) The potential application of finasteride for chemoprevention of prostate cancer. *Ann NY Acad Sci* **768**: 163–169

Keetch DW, Catalona WJ and Smith DS (1994) Serial prostatic biopsies in men with persistently elevated serum prostate specific antigen values. *J Urology* **151**: 1571–1574

McConnel JD, Bruskewitz R, Walsh P, Andriole G, Lieber M, Holtgrewe LH, Albertson P, Roehborn CG, Nickel JC, Wang DZ, Taylor AM and Waldstrelcher J (1998) The effect of finasteride on the risk of acute urinary retention and the need for surgical treatment among men with benign prostatic hyperplasia. *N Engl J Med* **338**: 557–563

Niesel T, Breul J, and Hartung R (1997) Diagnostic value of additional systematic prostate biopsies in patients undergoing transurethral resection of the prostate. *Urology* **49**: 869–874

Raviv G, Janssen T, Ziotta AR, Descamps F, Verhest A and Schulman CC (1996) Prostatic intraepithelial neoplasia: influence of clinical pathological data on the detection of prostate cancer. *J Urol* **156**: 1050–1055

Thompson IM, Coltman CA and Crowley J (1997) Chemoprevention of prostate cancer: The Prostate Cancer Prevention Trial. *Prostate* **33**: 217–221

Tsukmoyo S, Akaza H, Onozawa M, Shiral T and Ideyama Y (1998) A five-alpha reductase inhibitor or an antiandrogen prevents the progression of microscopic prostate carcinoma to macroscopic carcinoma in rats. *Cancer* **82**: 531–537

### Response to The effect of finasteride on the prostate gland in men with elevated serum prostate-specific antigen level

### Sir

The letter by Dr Coltman raises a number of important issues regarding our recent paper (Cote et al, 1998). As the first group to propose that 5α-reductase inhibitors might prove efficacious in prostate cancer prevention (Ross et al, 1992), we are well aware of the rationale for the potential efficacy of finasteride in preventing prostate cancer. We began our study fully expecting to observe beneficial effects on prostate cancer. Unfortunately we did not.

Although, we, too, were somewhat surprised by the low incidence of prostate cancer in the observational arm at the 1-year follow-up biopsy, we doubt that Coltman would have found acceptable a trial utilizing possibly inappropriate historical controls. The notion that you can change an observation in a trial and modify statistical values is hardly novel, and belies the entire purpose of statistical testing. Coltman raises the issue that if the cancer detection rate had been 7/27 and 1/25 in the treated and untreated groups, the *P*-value of the difference would be 0.051. Although this does not reach ‘statistical significance’, we would have considered such a difference to be very worrisome.

Coltman then states that if the patients with PIN are removed, the difference in cancer detection rates is meaningless. Two points can be made: (1) a clear and important conclusion of our study is that the presence of PIN is a strong risk factor for developing a positive biopsy after finasteride treatment; (2) in men without PIN the effect of finasteride on the diagnosis of prostate cancer is ‘meaningless’ because of small numbers. However, the much larger study referred to by Coltman of McConnel et al (1998) did not find a protective effect of finasteride either. In the latter trial, a total of 3040 men were randomized to receive finasteride or placebo for the treament of symptoms of benign prostatic hyperplasia. Treatment was for 4 years; in order to monitor for prostate cancer, 645 men underwent prostate biopsies during the study (325 men in the finasteride group and 320 men in the placebo group). The incidence of prostate cancer was 5% in each group, indicating no reduction in prostate cancer risk in a large population of lower risk men undergoing long-term treatment with finasteride.

Coltman repeatedly points to the small size of our trail. However, we were faced with the serious dilemma in the interim analysis of our study, that we might be causing harm. We decided that we could not justify continuing to accrue additional subjects.

Our trial, while small, remarkably provides the only data of any kind to date on the biological effects of finasteride on human prostate tissue. We strongly feel that studies such as ours are essential before embarking on large scale, long-term multi-institutional trials in healthy individuals. We undertook our study because of the dearth of any such relevant information.

As Chairman of the Southwest Oncology Group under whose auspices the US National Prostate Cancer Prevention Trial is being conducted, we believe the results of our study merit Dr Coltman’s attention, and should not be dismissed on dubious statistical grounds.

*RK Ross, MC Pike, E Skinner and RJ Cote*
*University of Southern California, School of Medicine,*
*1441 Eartlake Avenue, Los Angeles, CA 90033-0800, USA*

#### REFERENCES

Cote RJ, Skinner EC, Salem CE, Mertes SJ, Stanczyk FZ, Henderson BE, Pike MC and Ross RK (1998) The effect of finasteride on the prostate gland in men with elevated serum prostate-specific antigen levels. *Br J Cancer* **78**: 413–418

McConnel JD, Bruskewitz R, Walsh P, Andriole G, Lieber M, Holtgrewe LH, Albertson P, Roehbom CG, Nickel JC, Wang DZ, Taylor AM and Waldstrelcher J (1998) The effect of finasteride on the risk of acute urinary retention and the need for surgical treatment among men with benign prostatic hyperplasia. *New Engl J Med* **338**: 557–563

Ross RK, Bernstein L, Lobo RA, Shimizu H, Stanczyk F, Pike MC and Henderson BE (1992) 5-alpha reductase activity among Japanese and US white and black males. *Lancet* **339**: 887–890

### Platelets and VEGF blood levels in cancer patients

### Sir

It was with great interest that we read the letter to the Editor from Vermeulen et al (1999) regarding the accuracy of the measurement of vascular endothelial growth factor (VEGF) serum levels (VEGF-SL) and VEGF plasma levels (VEGF-PL) in cancer patients (Vermeulen et al, 1999). We have measured about 1000 serum samples for VEGF and should thus like to comment on their observations based on our findings:

1. We agree with Vermeulen et al that interindividual and intra-individual fluctuations of serum VEGF levels can, at least in part, be explained by variations of blood platelet counts. Another factor that may contribute to the variability of serum VEGF levels is the platelet volume. This notion is substantiated by our findings from patients receiving myeloablative chemotherapy. In these patients, screening of VEGF-SL was begun prior to the platelet nadir ($< 20$ G $l^{-1}$) and continued until the platelet counts had been recovered. VEGF was measured in 140 serum samples and 54 corresponding plasma samples by enzyme-linked immunosorbent assay (ELISA), essentially as described by Vermeulen et al. In line with their results, we found a striking correlation between peripheral blood platelet counts and absolute values of VEGF-SL ($r = 0.8$; $P < 0.001$), but not with VEGF-PL. Like Vermeulen and colleagues, we noted a broad inter-individual and intra-individual variability of VEGF-SL values. Thus, we hypothesized that VEGF-SL may not only depend on blood plaletet counts but also on the plaletet size. Platelets freshly released from the bone marrow following myeloablative chemotherapy may differ in size from those produced during a steady-state of myelopoiesis. To test this hypothesis, we compared the mean platelet volumes (MPV) prior to the platelet nadir with the MPV during the time of platelet reconstitution by using an electronic particle counter. In peripheral blood, the MPV before the platelet nadir indeed proved to be significantly lower than afterwards. Even if VEGF-SL values were corrected for the actual blood platelet count ($VEGF^{PLT}$ = VEGF-SL $\times 10^6$ platelets/actual blood platelet count), $VEGF^{PLT}$ values prior to the platelet nadir were significantly lower than those measured afterwards (Figure 1).
2. In vivo, platelet activation or platelet destruction causes major increments in VEGF blood levels: we detected high VEGF levels in both serum and plasma samples indicating a massive release of VEGF during intravasal platelet destruction in a patient with thrombotic-thrombocytopenic purpura.
3. Tumour cells and/or blood platelets may be the major sources of VEGF in cancer patients, particularly in metastatic disease. In fact, the majority of cancer patients with metastatic disease show elevated VEGF levels in serum (Kraft et al, 1999). Moreover, increasing VEGF serum levels may herald tumour relapse or progression. In a patient with breast cancer undergoing adjuvant high-dose chemotherapy, we noted a rapid

© 1999 Cancer Research Campaign
